# Primary healthcare professionals’ perspectives on mobility limitation assessment and predictive model optimization for community-dwelling older adults: a qualitative research

**DOI:** 10.3389/fpubh.2026.1905866

**Published:** 2026-08-31

**Authors:** Linbing Chen, Ye Ouyang, Chunyu Cheng, Xiyi Liu, Yanfei Xiang, Liping Wang

**Affiliations:** 1 School of Nursing, Guangdong Medical University, Dongguan, China; 2 Songshan Lake Community Health Service Center, Dongguan, China

**Keywords:** older adults, community, mobility limitation, primary healthcare, qualitative research

## Abstract

**Background:**

Mobility limitation is an important public health challenge faced by ageing populations. However, standardized assessment tools remain lacking in primary care practice in China, and existing predictive models still face substantial implementation gaps in clinical translation. This qualitative study aimed to explore frontline primary healthcare professionals’ perceptions of current assessment practices and to provide recommendations for optimizing and clinically translating related predictive models.

**Methods:**

A descriptive qualitative study was conducted in January 2026 among 15 frontline primary healthcare professionals in Dongguan City, China. Participants were recruited using maximum variation purposive sampling supplemented by snowball sampling. Semi-structured face-to-face interviews were analyzed using thematic analysis with NVivo 15.

**Results:**

Interviews with 15 community healthcare professionals (general practitioners, community nurses, and public health physicians) identified barriers, facilitators, and specific requirements for clinical implementation. Four principal themes emerged: (i) the current practice of assessment and perceived clinical value; (ii) feedback on model optimization and visions for diversified application; (iii) multidimensional barriers to clinical translation of the model; and (iv) creating a closed-loop support system with the older adults at the center.

**Conclusion:**

The findings show that primary healthcare professionals highly value assessment of mobility limitation, yet their assessment practices are inconsistent, and multiple barriers impede clinical translation. Future efforts should prioritize development of localized, intelligent, and cost-effective models and the establishment of a closed-loop management pathway spanning risk warning through follow-up.

## 1 Introduction

By the end of 2025, people aged 65 and older comprised 15.9% of China’s population, marking the country’s entry into a phase of advanced aging (1). At the same time, chronic disease and age-related physical deterioration have become more noticeable and create a significant burden on families and the health care system (2). The World Health Organization’s “Decade of Healthy Ageing 2021-2030” highlights the importance of maintaining functional abilities in older people as they grow older (3). Functional capacity is an individual’s capabilities within the context of their environment. Intrinsic ability includes locomotor capacity, which is the joint function, muscle strength, balance and endurance. Mobility is the actualization of locomotor capacity in an environment and mobility limitation occurs when disease or physical deterioration impedes movement and lower activity levels (4).

Mobility limitation is commonly assessed with the Short Physical Performance Battery(SPPB). Developed by Guralnik et al. (5), the SPPB comprises three components: balance tests, gait speed tests, and chair-stand tests. Each component is scored from 0 to 4, resulting in a total score ranging from 0 to 12. Higher scores indicate better physical performance. A total score of <10 was considered indicative of mobility limitation. Mobility limitation is a common manifestation of functional decline in older adults, with both prevalence and incidence remaining high. A systematic review found that the prevalence of mobility limitation ranged from 58.1 to 93.2%, while the incidence ranged from 23 to 53.7% (6). In China, related studies have reported a standardized prevalence of 30.4% and a 2-year incidence of 18.1% (7). Mobility limitation is associated with increased risks of cardiovascular disease and all-cause mortality (8, 9), and may further lead to poorer quality of life and greater psychological burden by limiting social participation in later life (10, 11). Among hospitalized older adults, it may also progress to adverse outcomes such as disability and contribute to increased healthcare burden (9, 12). Therefore, maintaining mobility is important not only for preserving physical function, but also as a fundamental prerequisite for active ageing.

In China, the Free Health Examination under the National Essential Public Health Services (NEPHS) Program for Older Adults Aged 65 and above is an important public health initiative for monitoring and maintaining the health of older adults. However, this program does not currently include standardized mobility limitation assessment among older adults, such as the SPPB (13). A review of the literature showed that existing prediction models for mobility limitation still have several important limitations. Some models use subjective self-reported outcomes (14), some were developed in specific disease populations (15), and others incorporate the SPPB as a predictor (16). These limitations make existing models unsuitable for large-scale community screening in China. To address this gap, our team developed a novel risk prediction model for mobility limitation based on the China Health and Retirement Longitudinal Study (CHARLS) database. Predictor selection was aligned as closely as possible with the routine clinical indicators collected in the Free Health Examination under the NEPHS Program for Older Adults Aged 65 and above. After feature selection, six core predictors were included in the final model: age, handgrip strength, cognitive function, ADL/IADL disability, and living alone. Among the candidate algorithms, Naïve Bayes achieved the best overall performance. However, its low recall (0.364) and limited clinical net benefit indicate that it is still not suitable for real-world implementation. A full comparison of model predictors and performance is provided in Supplementary material 1. The transition of a model from theoretical construction to practical clinical application requires not only a sound quantitative research design, but also full consideration of the perspectives of primary healthcare professionals. Although algorithm optimization is indeed one of the important means to improve model performance, it is difficult to solve complex clinical problems solely through technical optimization. In this context, the perspectives of primary healthcare professionals can precisely inform the direction of model iteration and its clinical translation.

Therefore, based on the previous research of our team on the prediction model, this study adopted a descriptive qualitative research design. From the clinical perspective of primary healthcare workers, it aims to explore the current status of mobility limitation assessment among community-dwelling older adults, and to discuss in depth the clinical optimization strategies for the model (including the supplementation of predictive indicators and the presentation form of tools), as well as the facilitators and barriers affecting its community promotion. The ultimate goal is to provide empirical evidence to inform the localization and iterative refinement of this model for clinical translation.

## 2 Methods

### 2.1 Study design

This study used a qualitative descriptive design and semi-structured interviews to capture frontline healthcare workers’ perspectives and suggestions. To minimize individual interpretation bias and ensure analytical rigor, two independent researchers (LC and YOY) with distinct background experiences conducted thematic analysis to distill core themes (17). The study followed the Consolidated Criteria for Reporting Qualitative Research (COREQ) checklist to ensure methodological rigor and transparency (18). The COREQ checklist is provided in Supplementary material 2.

### 2.2 Population

This study adhered to the principle of maximum variation sampling, employing both purposive sampling and snowball sampling to recruit frontline community healthcare workers, while fully accounting for variations among participants in terms of age, gender, educational background, job position, years of service, and experience in conducting health examinations for older adults (19). Differences among participants in terms of age, sex, educational level, professional role, years of work experience, and years of experience in conducting health examinations for older adults were fully considered. In January 2026, frontline team members involved in the Free Health Examination for Older Adults Aged 65 and above under the NEPHS Program were recruited as study subjects from a community health service center and its affiliated stations in Dongguan City, Guangdong Province, China.

Based on recommendations from colleagues and department heads, potential study subjects were preliminarily identified. Researchers provided detailed written study information to the potential subjects, who were formally enrolled in the study upon signing an informed consent form. Inclusion criteria: (i) Full-time employment at a community health service center that implements the Free Health Examination for Older Adults Aged 65 and Above under the National Essential Public Health Services (NEPHS) Program; (ii) Practice category of general practitioner, public health physician, or community nurse; (iii) Direct participation in this examination program for ≥ 2 years; and (iv) Voluntary participation in the study and signing of an informed consent form.

The sample size was set by data saturation; recruitment stopped when no new themes or perspectives emerged during analysis (20). Of 17 eligible participants, 15 consented, yielding an 88.2% response rate, and none withdrew. To preserve privacy, participants’ names were replaced with codes: D1–D7 for general practitioners, N1–N4 for community nurses, and P1–P4 for public health physicians. The study was approved by the Institutional Review Board of the Songshan Lake Community Health Service Center, Dongguan (Approval No. 202509).

### 2.3 Data collection

Based on prior literature and predictive-model results, we developed a semi-structured interview guide. To ensure logical coherence and content validity, two associate professors of nursing reviewed and revised the initial outline. We then conducted preliminary interviews with two primary healthcare workers to evaluate question clarity and feasibility. As no substantive changes resulted from these pilot interviews, we included the two pilot transcripts in the formal analysis.

The interview guide comprised three thematic domains: (i) assessment practices and perceptions, covering the current status of mobility limitation assessment among community-dwelling older adults and healthcare professionals’ views on its value; (ii) model evaluation and optimization recommendations, soliciting suggestions for supplementing and refining the core predictors of the preliminary predictive model; and (iii) application barriers and implementation prospects, exploring factors that hinder clinical translation of an idealized assessment tool in primary care, as well as potential application models and promotion strategies. The full interview guide is presented in Table 1.

**Table 1:** Semi-structured interview guide.

Part 1: Assessment practices and perceptions
*[Interviewer prompt: Prior to this section, the interviewer briefly introduces the WHO definitions of ‘mobility’ and ‘mobility limitation,’ and presents the Short Physical Performance Battery (SPPB) based on WHO guidelines as a reference point.]*
Q1. During routine health examinations or management, do you pay attention to the “mobility” of older adults? What specific signs or methods do you rely on to assess it?
Q2. Do you believe it is necessary to incorporate a standardized mobility assessment into the routine free health examination program for adults aged 65 and older? How feasible is this in current practice?

Part 2: Model evaluation and optimization
*[Interviewer prompt: Prior to this section, the interviewer presents the construction process and preliminary findings of the study’s predictive model using standardized visual aids (*e.g.*, model diagrams) and scripted verbal explanations to ensure consistency across all interviews.]*
Q3. In your prior clinical work, have you used predictive models or risk-assessment tools? Could you describe your experience with them and share your views on these tools?
Q4. Given the limitations of our preliminary model, do you consider it necessary to optimize the model using local real-world health examination data? Why?
Q5. Based on your clinical frontline experience, what additional health examination indicators or variables should be incorporated into the predictor pool?
Q6. If the model performs well, which presentation format would you prefer: a mobile mini-program, a paper scoring sheet, or integration into an existing system? Please state your preference and the reasons.

Part 3: Application barriers and future prospects
Q7. If a well-performing predictive model were implemented at your center, what factors would facilitate its adoption and what barriers might impede it?
Q8. Do you have any additional insights, concerns, or suggestions regarding the mobility assessment or prediction models discussed today?

LC conducted data collection through face-to-face interviews held in a quiet, private room at each participant’s medical facility. The primary interviewer (LC) was a male master’s student in nursing who had training in qualitative research. Interviews were arranged at times convenient for participants to minimize disruption to their clinical duties. Before every interview the researcher restated the study’s goals and the voluntary nature of participation, as well as the consent to audio recording, and that the participant may leave the interview at any time without repercussions. The researcher then obtained basic demographic information. The formal interview was conducted using the interview guide with follow up, clarification, and paraphrasing used to promote key information. Interviews lasted 20–33 min. All sessions were recorded on a secure mobile phone with encrypted recording software and nonverbal behavior was documented at the same time. Transcripts were real-time created and reviewed and approved by the participants on location. All electronic data were kept on password protected computers and on encrypted hard drives only available to the research team.

### 2.4 Data analysis

The transcripts were checked by two researchers (LC and YOY), and cross-checked with the original recordings. Thematic analysis was conducted using Braun and Clarke’s six stages of analysis and used a bottom-up approach to coding the data which was organised using NVivo 15 (17). Two dimensions of the current state of assessment of mobility limitation in older community-dwelling persons and recommendations for enhancing predictive models were coded line by line by LC and YOY separately. LC was involved in the development and evaluation of the preliminary predictive model and employed a bracketing strategy during qualitative analysis to actively set aside prior modeling background and preconceived assumptions. YOY had no prior involvement in machine learning-related work, and her independent perspective helped balance potential confirmation biases that LC might hold. Additionally, LC and YOY also wrote analytical memos to document coding rationales and to reflect on the researchers’ assumptions and positionality to increase the transparency and credibility of the study. Data was coded independently and checked for discrepancies and consensus was reached; if discrepancies arose, they were judged by LW, Associate Professor of Nursing. The constant comparison method was used to group codes into initial themes. Conceptual boundaries of each theme were clarified and naming of themes was made to be congruent with clinical practice through repeated iterations and continual refinement. Then, representative quotations were chosen to represent each theme. The quotations were first translated into English by a researcher and then checked and refined by a professional translation service to ensure that the meaning of the original Chinese was conveyed without losing its nuances.

## 3 Results

### 3.1 Participant characteristics

There were 15 primary healthcare workers. The age range of the participants was 30–49 years, their years in practice ranged from 6–22 years, and the number of years performing health assessments in older adults ranged from 2–18 years. They included 3 men and 12 women; 14 who were educated to undergraduate level and 1 who was educated to postgraduate level; 7 general practitioners, 4 community nurses and 4 public health physicians. There was limited knowledge with regards to predictive models among some of the participants. Table 2 shows demographic information.

**Table 2:** Characteristics of interview participants.

Participant ID	Age (y)	Gender	Educational background	Total experience (y)	Older adult exams (y)
General practitioners (*n* = 7)					
D1	40	Male	Undergraduate	20	18
D2	37	Female	Postgraduate	11	2
D3	49	Male	Undergraduate	20	15
D4	43	Female	Undergraduate	16	15
D5	30	Male	Undergraduate	6	3
D6	35	Female	Undergraduate	8	5
D7	43	Female	Undergraduate	17	17
Community nurses (*n* = 4)					
N1	42	Female	Undergraduate	22	5
N2	44	Female	Undergraduate	21	9
N3	36	Female	Undergraduate	15	3
N4	33	Female	Undergraduate	10	4
Public health physicians (*n* = 4)					
P1	38	Female	Undergraduate	15	12
P2	39	Female	Undergraduate	14	3
P3	36	Female	Undergraduate	12	4
P4	39	Female	Undergraduate	15	10

y, years; Older adult exams, years of experience in performing free health examinations for adults aged ≥65 years.

### 3.2 Theme

Four major themes and eleven subthemes were identified using the thematic analysis. The themes were: (i) the current practice of assessment and perceived clinical value; (ii) feedback on model optimization and visions for diversified application; (iii) multidimensional barriers to clinical translation of the model; and (iv) creating a closed-loop support system with the older adults at the center. Table 3 gives additional information.

**Table 3:** Themes and subthemes identified through thematic analysis.

Themes	Sub-themes
(i) The current practice of assessment and perceived clinical value	The absence of attention and assessment practices
	Broad appreciation of the potential of assessment
	Pre-conditions for integrating into regular health checks
(ii) Feedback on model optimization and visions for diversified application	Practical considerations and perceived value of model refinement
	Recommendations for incorporating additional key predictors
	Strategies for diversifying and adapting tool formats
(iii) Multidimensional barriers to clinical translation	Intrinsic barriers related to model attributes
	Resource and policy constraints at the institutional level
	Challenges concerning the compliance of the target population
(iv) Creating a closed-loop support system with the older adults at the center	Developing a closed-loop management pathway: assessment, intervention, and follow-up
	The value of longitudinally tracking individual mobility trajectories

#### 3.2.1 The current practice of assessment and perceived clinical value

This theme shows that community healthcare providers value mobility assessment in older adults but have great barriers to the practice of mobility assessment in clinical practice. Respondents recognized the value of these assessments when it comes to public health for an aging population, but standardized evaluations were not often used as a part of routine clinical care or health exams. All this will need to be complemented by addressing the shortage of primary care staff, the limited time for examination, and simplified tools for mobility assessment, as well as national policies and guidance, for the effective integration of mobility assessment into free health checks to become a reality for older adults. The theme consists of three subthemes: (a) the absence of attention and assessment practices; (b) broad appreciation of the potential of assessment; and (c) pre-conditions for integrating into regular health checks.

##### 3.2.1.1 The absence of attention and assessment practices

The interviews indicated that mobility limitation is relatively common among community-dwelling older adults; however, systematic assessment of this problem has not been routinely integrated into clinical practice or free health screenings. Primary health care workers typically lack standardized protocols and rely on visual gait observations, informal use of assistive devices, or indirect judgments from activities-of-daily-living assessments, and they rarely employ objective, quantitative tools. One public health physician commented:

“Generally, if they can walk in normally… we assume they are fine. But if they come in with a cane or need to be supported by someone, their mobility is definitely poor…” (P2).

Primary healthcare professionals’ assessment of mobility mainly relied on experiential judgment. As one general practitioner stated:

“We mainly have their mobility assessed by evaluating their activities of daily living.” (D4).

##### 3.2.1.2 Broad appreciation of the potential of assessment

At the cognitive level, participants strongly affirmed the clinical value of mobility assessment. Driven by the dual forces of population aging and national public health policies, primary healthcare professionals generally considered it both necessary and of far-reaching significance to incorporate this assessment into the free health examination program for older adults. As one general practitioner emphasized:

“It is definitely necessary. Population ageing is becoming increasingly serious… This is not something that can be identified through a one- or two-minute inquiry during a physical examination… If it became a mandatory requirement, more people would be screened.” (D7).

##### 3.2.1.3 Pre-conditions for integrating into regular health checks

Most participants remained skeptical about whether mobility assessment could actually be carried out in busy community settings. Shortages in primary care human resources and heavy workloads were regarded as the primary constraining factors, and assessment procedures that were time-consuming were considered particularly difficult to implement during centralized health examinations. They believed that the clinical implementation of this assessment should be guided by policy support and should also meet conditions such as ease of operation, manageable costs, and active cooperation from older adults. Reflecting on this clinical reality, one general practitioner shared:

“For now, the process takes too long and may not be practical.” (D3).

A community nurse discussed the acceptance among Older adults and the implementation costs, stating:

“If it is simple and effective… and if older adults are willing to cooperate, then I think it could be implemented.” (N2).

A public health physician pointed out from a policy perspective:

“Our examination content follows the third edition of the national basic public health service standard. If the national experts decide to include this… then we will implement it accordingly.”(P1).

#### 3.2.2 Feedback on model optimization and visions for diversified application

The second theme centered on how participants thought the predictive model could be refined and eventually put to use in community settings. In view of the timeliness of the existing data and the model’s current evaluation results, community healthcare professionals indicated that the model still has the potential to be further explored using local data. At the same time, they expressed expectations that smart assessment tools could meaningfully empower primary care work. Participants believed that a predictive model with real clinical value should not only demonstrate high accuracy and sensitivity, but also that its final implementation format should also align with the information-based workflow needs of primary care and the diverse characteristics of older adults. This theme comprises three sub-themes: (i) practical considerations and perceived value of model refinement; (ii) recommendations for incorporating additional key predictors; and (iii) strategies for diversifying and adapting tool formats.

##### 3.2.2.1 Practical considerations and perceived value of model refinement

Participants indicated that the preliminary predictive model still required further optimization, partly due to factors such as the timeliness of the data. From a clinical standpoint, a well-performing predictive model is valuable not only for individual health management but also for population-level prevention. Primary healthcare professionals hoped that screening tools with good sensitivity and ease of use could help identify high-risk individuals who tend to be overlooked during health examinations. This would be a valuable support for routine primary care practice. As one general practitioner stated:

“I think that from 2015 to now, over the past 10 years, changes in people’s nutrition and social structure have led to substantial changes in their physical condition… From a sociological perspective, identifying people with early mobility limitation and providing early intervention… is definitely very meaningful and valuable. Especially in the context of population aging… from a research perspective, I think if there is a method that can simply help community colleagues conduct initial screening and identify those at risk, and if it is simple and easy to implement, then the easier it is to carry out, the more meaningful it becomes.” (D1).

##### 3.2.2.2 Recommendations for incorporating additional key predictors

The interviews revealed that although most participants did not identify specific predictors, some participants who were familiar with predictive models offered targeted suggestions from a clinical perspective. They suggested that attention should be paid to the role of sensory function, such as vision, chronic comorbidities, such as chronic obstructive pulmonary disease (COPD), and objective biochemical indicators in prediction. One general practitioner explained:

“…Vision… If his vision is very poor and he doesn’t exercise for a long time, his mobility will naturally be limited. …For example, COPD…”

(D1).

In addition, some respondents suggested leveraging existing blood test data to further improve the model’s predictive performance without adding an additional evaluation burden.

“There are also many test results from the blood draw during a senior health checkup.” (P1).

##### 3.2.2.3 Strategies for diversifying and adapting tool formats

Based on the ideal model outlined above, respondents argued that the predictive tool should use multiple presentation formats to balance primary care efficiency with accessibility for diverse older adult groups: primarily embedded in information systems, with mobile devices and paper-based materials as supplements. One public health physician described the efficiency gains:

“I think mobile mini-programs make it easier for our residents to communicate with doctors… A plugin integrated into the physical exam system would be even better and more convenient—it would eliminate the need for repetitive work… It would be a bit smarter and save time and effort.” (P4).

Another public health physician, focusing on accessibility for older adults, stressed the need for coexisting formats:

“Some of our residents are highly educated and skilled with smartphones, so they can use mini-programs; but others have very low levels of education or are even illiterate, so we may need to provide one-on-one assistance—we may have to use a variety of methods.” (P1).

#### 3.2.3 Multidimensional barriers to clinical translation

This theme highlights practical barriers to translating an idealized risk prediction model for mobility limitation from theory into primary care. Barriers cluster in three interrelated dimensions: technical attributes of the predictive model, institutional resource and policy constraints, and acceptance by the target population. Participants emphasized that implementing assessment tools in primary care requires practical, cost-effective design, top-down policy and resource support, and careful consideration of older adults’ physiological and cognitive limitations. Without subsequent intervention resources and policy drivers, a isolated screening process may struggle to achieve its intended goals. This theme comprises three sub-themes: (i) intrinsic barriers related to model attributes; (ii) resource and policy constraints at the institutional level; and (iii) challenges concerning the compliance of the target population.

##### 3.2.3.1 Intrinsic barriers related to model attributes

Participants’ acceptance of the predictive model mainly depended on its practical utility. Whether the model’s predictions were reliable, whether it was easy to operate, and whether it involved substantial economic costs were important considerations in judging its feasibility for implementation in primary care. Overly cumbersome system login procedures or unclear charging standards could directly affect the promotion and application of the model at the primary care level. As one general practitioner briefly summarized:

“First, how effective is it… second, how easy is it to operate?” (D1).

A community nurse expressed her concerns:

“Will this require a fee? …And then there is the reliability of your data… also, what tools would need to be used, such as your dedicated app, and whether it is easy to log in…” (N3).

##### 3.2.3.2 Resource and policy constraints at the institutional level

Participants indicated that the successful implementation of the model would depend heavily on top-down policy directives and dedicated human resource support. More importantly, they emphasized that without effective linkage to subsequent intervention resources, isolated screening would be highly likely to become a mere formality, adding to the workload of primary care while failing to realize its intended public health value.

A community nurse stated directly:

“Whether it can be promoted and implemented, I think, depends on directives from above… If it becomes a requirement at the municipal level, I think it could still be rolled out comprehensively.” (N1).

One general practitioner added from the perspective of human resource burden:

“If we have to assign additional staff specifically to screen and assess these older adults, it will definitely increase the workload to some extent.” (D5).

Another general practitioner expressed concern about follow-up after screening:

“…whether those who test positive will receive adequate support afterward… whether there are sufficient interventions to help them…” (D1).

##### 3.2.3.3 Challenges concerning target population compliance

Participants reported that cognitive biases in older adults, together with reduced physiological tolerance, substantially hinder data collection. Residents often do not perceive their mobility limitations as urgent, and factors such as fasting-related fatigue and memory lapses during physical examinations can produce poor cooperation or inaccurate responses, which in turn undermine the model’s early-warning accuracy. A public health physician stated:

“Because residents currently have very low awareness of mobility limitation in older adults… when we tell them that a test needs to be done, their cooperation may be very poor…” (P3).

A general practitioner further described the difficulties encountered during health examinations:

“During health examinations for older adults, the time allocated to each person is actually quite limited. If you need to collect data and ask many questions, and the older adults cannot remember clearly, much of the data may be incomplete. If you do not spend enough time, the accuracy of the data may also be low.” (D6).

#### 3.2.4 Constructing a person-centered, closed-loop support system

This theme reveals the long-term vision and ideal service model for the practical application of the predictive model in primary care. The interviews revealed that risk alerts alone do not represent the ultimate goal of health management. Instead, it is only through building a continuous, older adult-centered care system that the clinical value of assessment tools can be truly realized. Participants emphasized not only the need for a horizontal, closed-loop pathway covering screening, assessment, intervention, and follow-up, but also the importance of using health records for ongoing, individualized longitudinal monitoring. This theme comprises two sub-themes: (i) developing a closed-loop management pathway: assessment, intervention, and follow-up; and (ii) the value of longitudinally tracking individual mobility trajectories.

##### 3.2.4.1 Developing a closed-loop management pathway: assessment, intervention, and follow-up

Participants emphasized that assessment tools should be deeply integrated with follow-up services to establish a whole-cycle health management continuum. Screening results for mobility limitation in older adults may lose their clinical value if they are not accompanied by professional interpretation and targeted intervention. A closed-loop mechanism that integrates risk warning, professional interpretation, precision intervention, and dynamic follow-up is essential to realizing the full clinical value of the predictive model. A community nurse observed:

“…Once you include it in the assessment, you must explain to residents what your next steps will be and how you plan to proceed…You need to provide them with a report interpreting the results and outline your follow-up actions. Conducting a screening must yield results, but what happens after that is what matters most.” (N4).

A general practitioner made a similar point:

“Even if the test comes back positive or shows something, you still need a plan for intervention. What kind of intervention measures do you have in place…?” (D2).

##### 3.2.4.2 The value of longitudinally tracking individual mobility trajectories

The screening of the respondents was also done on a cross-sectional basis, and the respondents also highlighted the predictive model’s support for longitudinal monitoring. They think it is possible to track both changes over time in individual’s mobility and identify potential risks earlier than by one single cross sectional assessment and thus, secure targeted prevention, by using the free health checks that are conducted for Older adults every year. A GP explained it thus:

“In this model, I think it’s important to have a retrospective component to compare this year to last year… a change within a specific population over time… When looking at a population, his performance this year and last year could be within the normal range, but his own mobility has decreased…” (D1).”

## 4 Discussion

Our analysis yielded four core themes: (i) The current practice of assessment and perceived clinical value; (ii) Feedback on model refinement and visions for diversified application; (iii) Multidimensional barriers to clinical translation; and (iv) Creating a closed-loop support system with the older adults at the center. Overall, primary healthcare professionals face a contradiction between high value recognition and low standardized implementation in the assessment of mobility limitation. Regarding the optimization and translation of the predictive model, participants proposed pragmatic suggestions, including localized iteration, diversified delivery formats, and supporting policies and resources. On this basis, the study further clarified that shifting from isolated screening to full-cycle closed-loop management encompassing assessment, intervention, and longitudinal tracking is a key pathway for realizing the clinical value of the assessment tool.

In terms of the current assessment landscape, this study found a pronounced supply–demand paradox among community healthcare professionals regarding the assessment of mobility limitation in older adults. On the one hand, community healthcare professionals highly recognized, at the cognitive level, its value in preventing falls, improving older adults’ well-being, and generating broader social benefits. On the other hand, clinical practice remains marginalized, with assessments largely relying on non-quantitative, rough judgments. This is highly consistent with the findings of Yang et al. (21), which showed that although general practitioners generally support early screening for dementia, assessment work remains difficult to fully implement in clinical practice. Similarly, Sideman et al. (22) also revealed this contradictory situation in primary care, where strong recognition of the importance of assessment coexists with practical barriers. The strong value recognition among healthcare professionals may stem from the deep internalization of preventive concepts embedded in the general practitioner gatekeeper model (23). It may also be driven by the national healthy ageing strategy, through which healthcare professionals have observed the practical benefits of active ageing in maintaining functional ability and improving quality of life among older adults (24). However, the translation of this value recognition into clinical practice is severely constrained by two major barriers: the lag in standardized guidelines and excessive workforce burden. Chinese scholars have previously explored older adults’ intrinsic capacity using the WHO ICOPE framework (25). However, it was not until 2024 that a national expert consensus formally recommended a standardized mobility assessment tool—the SPPB (26). As a result, such assessments have long been absent in primary care settings. Community healthcare workers account for only 5.1% of the total national health workforce (27), yet they are required to undertake a wide range of primary healthcare responsibilities, including the National Basic Public Health Services. Moreover, more than 80% of local primary healthcare professionals work over 40 h per week (28). In the future, it is recommended that standardized assessment tools be incorporated into the performance evaluation items of primary public health services, while efforts should also be made to increase human resources. These measures may help healthcare professionals better carry out primary-level prevention and control of mobility limitation among older adults.

Interview results showed that the surveyed community healthcare professionals generally recognized the clinical significance of predictive models for mobility limitation. With social changes and greater data accessibility, participants felt it was time to localize and refine the predictive model based on the realities of their own communities. Although the predictive model based on the CHARLS database has limited sensitivity, this does not mean that the field lacks research value. In previous studies using CHARLS data to develop prediction models for older adults, some models demonstrated good performance, while others showed only moderate performance, suggesting room for further optimization (29). Other studies have shown that when predictive models are transferred from development datasets to new external data sources or real-world settings, they often experience reduced discrimination and calibration drift, thereby requiring local recalibration, model updating, or redevelopment (30, 31). As auxiliary decision-making tools, predictive models have been widely explored and, to some extent, validated in areas such as fall risk assessment and sarcopenia screening among older adults (32, 33). This is highly consistent with the expectations expressed by the interviewees in this study regarding the use of tools to empower primary care and safeguard the health of older adults. Currently, the NEPHS Program requires annual free health examinations for older adults, providing continuous, multidimensional, and readily accessible clinical examination data. If assessments of mobility limitation are further standardized and incorporated into the health examination process in the future, this would be more conducive to developing and validating community-adapted predictive models in primary care settings. The additional predictors mentioned by the interviewees, namely vision, COPD, and blood test results, may also be considered in future research to further explore the associations between variables in existing health examination data and mobility limitation. These suggestions may reflect participants’ reliance on clinically observable indicators, given their limited familiarity with the technical details of predictive models.

The interview results of this study suggest that, assuming the predictive performance of the model reaches an ideal level, primary care professionals’ expectations for predictive tools are not limited to accuracy. They place greater emphasis on whether such tools can save time and effort, minimize additional workload in high-throughput settings such as health examinations for older adults, and accommodate older populations affected by the digital divide, thereby enabling sustainable implementation. In previous studies, the translation vehicles for predictive models have mainly focused on static nomograms or dynamic web-based calculators/mini-programs, with some studies adopting a dual strategy combining graphical tools and digital tools (34–36). Against the overlapping backdrop of population ageing and digitalization, smart healthcare should not only improve efficiency but also pay attention to the accessibility and equity needs of older adults. Although diversified presentation formats have been considered to better align with the realities of community clinical practice, the translation process remains constrained by three sets of factors: the model itself, healthcare institutions, and the target population. Studies have shown that the main barriers to clinical translation include the lack of automatic data retrieval and reminder functions, which increases the workload of healthcare professionals; insufficient workforce capacity, performance incentives, and intervention resources, which make it difficult to establish a closed-loop screening pathway; and overly technology-centered model design, which neglects user adaptation (37, 38). Therefore, promoting translation requires system-level restructuring to achieve the organic integration of technological integration, resource coordination, and user adaptation. Predictive models developed in previous studies have primarily focused on risk prediction. However, healthcare professionals in the interviews further emphasized that model application should be integrated with a closed-loop management pathway encompassing assessment, intervention, and follow-up, while gradually exploring longitudinal tracking of individual locomotor capacity. Currently, most predictive model studies focus primarily on the stages of model development or validation (39), the assessment of mobility limitation among older adults, as well as subsequent diagnosis, intervention, and follow-up, remains relatively absent or is still at a fragmented exploratory stage in many healthcare institutions. Although the relevant expert consensus published in 2024 proposed several recommendations for multidisciplinary management, further improvement is still needed in terms of overall implementation and community-based management models that link primary care institutions with higher-level hospitals (26). In recent years, under the guidance of the National Health Commission, various regions have gradually established a closed-loop management pathway of “community screening, hospital diagnosis, and community follow-up” by incorporating cognitive function screening into free health examinations for older adults (40). A study conducted by Yang et al. (41) in Wisconsin showed that a community–hospital closed-loop management pathway is feasible and can produce health benefits. The longitudinal tracking of individual locomotor function mentioned in the interviews points to a direction for future research. This strategy can not only enable ultra-early warning but also provide objective evidence for the dynamic evaluation of intervention effects and subsequent adjustment of intervention plans, thereby promoting the transformation of mobility management in older adults toward individualized precision navigation.

This study also has several limitations. First, all interviewees were recruited from the same community health service center and its affiliated stations in Dongguan City. As a relatively economically developed region in Guangdong Province, Dongguan’s medical insurance and referral policies differ significantly from those in other regions, and there are also substantial disparities in primary healthcare infrastructure and workforce allocation between Dongguan and other cities in China. Therefore, the results of this study should be interpreted cautiously when applied to resource-limited settings. Nevertheless, this study adopted maximum variation sampling, covering different professional roles such as physicians, nurses, and public health physicians, and the interview data reached information saturation, giving the findings certain reference value. Second, this study focused on clinical healthcare professionals and did not include feedback from older adults. Given that the study is still at an exploratory stage, prioritizing the perspective of service providers at this stage is methodologically justified. Finally, as a qualitative research, this research lacks quantitative data from real-world implementation processes. However, identifying key pre-implementation barriers can provide valuable reference for future translational research.

## 5 Conclusion

This study adopted a descriptive qualitative research design to explore the current status of mobility limitation assessment among older adults, directions for optimizing predictive models, and clinical translation pathways from the clinical perspective of community healthcare professionals. The findings revealed a supply–demand paradox among primary care professionals, characterized by high value recognition but low clinical implementation of such assessments. At the same time, this study clarified that predictive models can be further iterated toward localization, intelligence, and low-burden application, and revealed that their clinical translation is subject to three systemic constraints: the inherent attributes of the model itself, the resources of healthcare institutions, and the level of cooperation among the target population. Based on these findings, this study proposes that future efforts should focus on constructing a closed-loop service pathway of “community screening–professional referral–precision intervention” and exploring individualized longitudinal tracking based on annual health examination data.

## Data Availability

The datasets used in this study are not publicly available due to privacy and confidentiality concerns involving human participants. De-identified data may be made available from the corresponding author upon reasonable request at wlp2012@gdmu.edu.cn.
