# Supplementary material for: Primary healthcare professionals’ perspectives on mobility limitation assessment and predictive model optimization for community-dwelling older adults: a qualitative research
Source: Front Public Health. 2026 Aug 31;14:1905866. doi: 10.3389/fpubh.2026.1905866 (PMC13572106; doi:10.3389/fpubh.2026.1905866)
Supplement: Supplementary file 1 [file Data_Sheet_1.PDF]

Table S1. Categorization and coding of predictor variables based on the Health Ecology Model

Level	Variable Name	Coding
**Individual biological determinants**		
	Age (years)	Continuous value
	Gender	Male=1, Female =0
	Education level	Illiterate=1, Primary school=2, Secondary school=3, College and above=4
	Hypertension	Yes=1, No=0
	Diabetes	Yes=1, No=0
	Chronic lung disease	Yes=1, No=0
	Heart disease	Yes=1, No=0
	Stroke	Yes=1, No=0
	Chronic pain	Yes=1, No=0
	History of falls	Yes=1, No=0
	Self-reported health status	Poor=1, Fair=2, Good=3
	BMI (kg/m^2)	Continuous value
	Handgrip strength (kg)	Continuous value
**Individual lifestyle behaviors**		
	Smoking status	Yes=1, No=0
	Drinking status	Yes=1, No=0
	Life satisfaction	Poor=1, Fair=2, Good=3
	Depression	Yes=1, No=0
	ADL disability	Yes=1, No=0
	IADL disability	Yes=1, No=0
	Cognitive function (score)	Continuous value
	Sleep duration (hours)	Continuous value

Table S1. Categorization and coding of predictor variables based on the Health Ecology Model

Level	Variable Name	Coding
**Social and community networks**		
	Marital status	Others=1, Married=0
	Living alone	Yes=1, No=0
	Social participation	Yes=1, No=0
**Living and environmental conditions**		
	Residence	Urban=1, Rural=0
**Macro-level policy environments**		
	Endowment insurance	Insured=1, Uninsured=0
	Health insurance	Insured=1, Uninsured=0

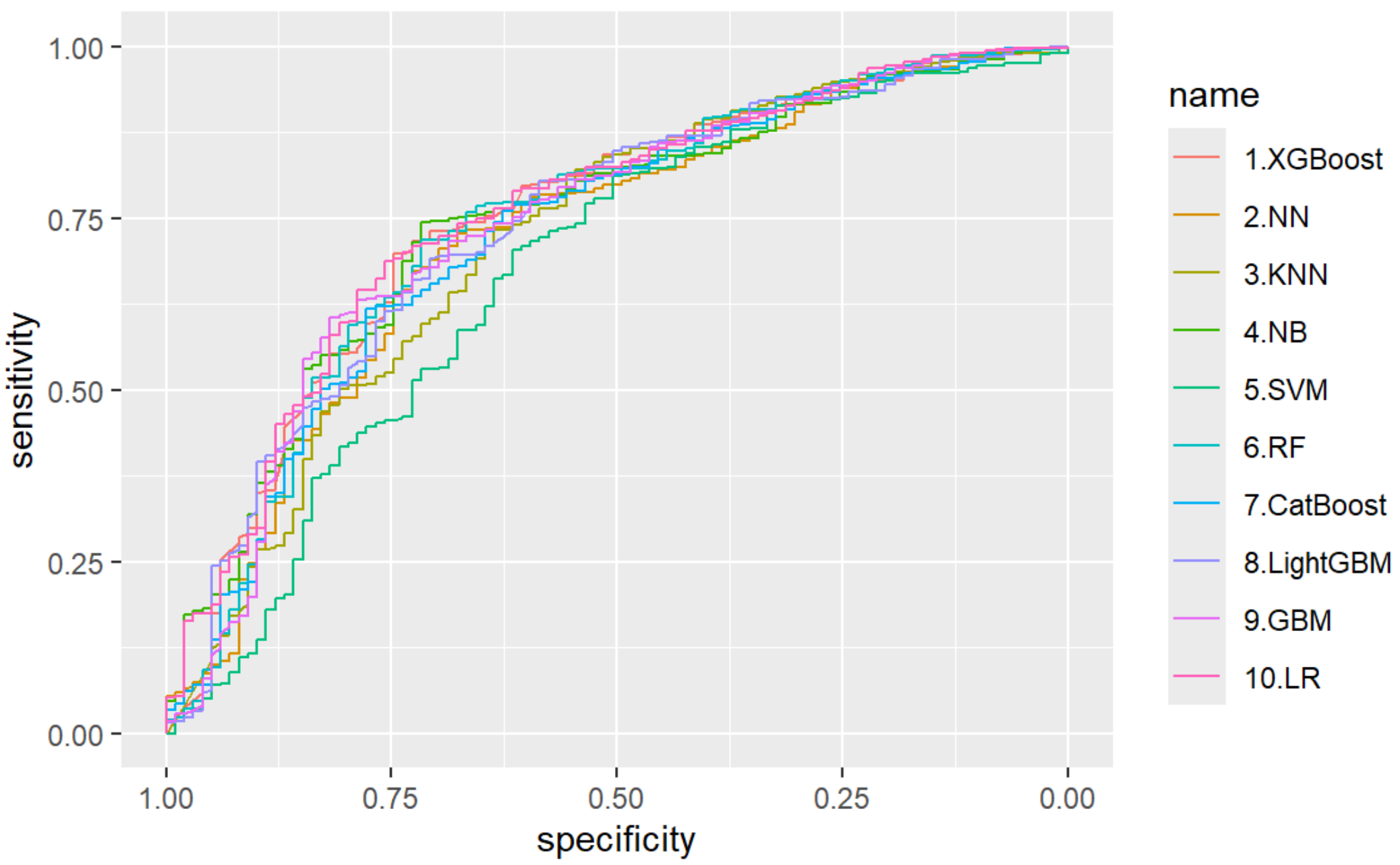

Figure 1 ROC curves comparison of different models

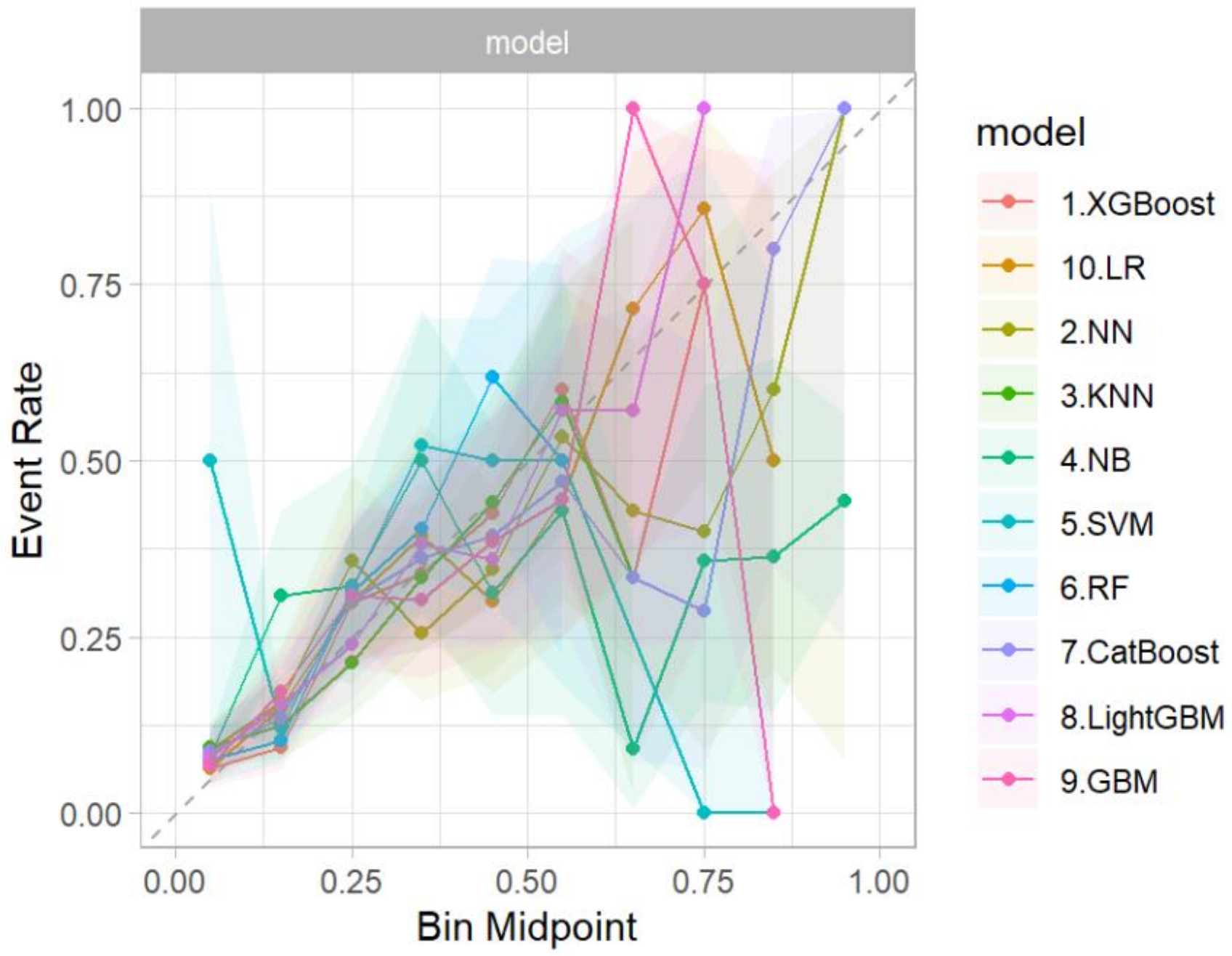

Figure 2 Calibration curves comparison of different models

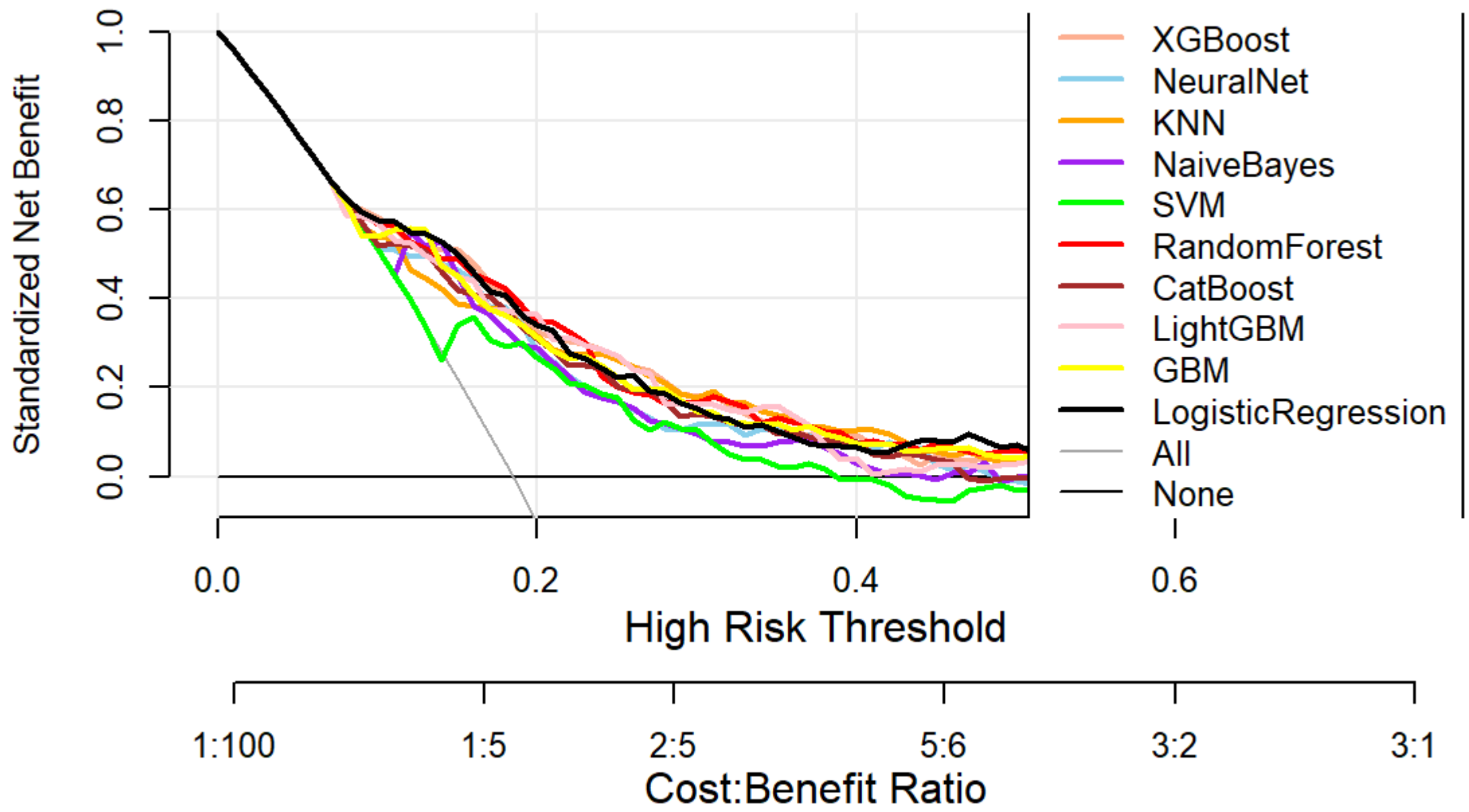

Figure 3 Decision curve analysis comparison of different models

Table S2. Performance metrics of candidate machine learning models in the training and testing datasets

Model	Dataset	AUC	Accuracy	Precision	Recall	F1-Score	Brier Score	Specificity	Negative Predictive Value
Logistic Regression	Training	0.782	0.832	0.611	0.239	0.344	0.122	0.966	0.850
	Testing	0.756	0.827	0.588	0.202	0.301	0.129	0.968	0.843
Random Forest	Training	0.854	0.834	1.000	0.096	0.175	0.114	1.000	0.831
	Testing	0.741	0.816	0.500	0.030	0.057	0.131	0.993	0.819
Naïve Bayes	Training	0.765	0.796	0.441	0.426	0.434	0.161	0.879	0.872
	Testing	0.744	0.773	0.379	0.364	0.371	0.177	0.865	0.857
Extreme Gradient Boosting	Training	0.833	0.848	0.763	0.252	0.379	0.112	0.982	0.854
	Testing	0.750	0.821	0.560	0.141	0.226	0.130	0.975	0.834
Categorical Boosting	Training	0.885	0.883	0.919	0.396	0.553	0.092	0.992	0.880
	Testing	0.729	0.814	0.486	0.182	0.265	0.135	0.957	0.838
Light Gradient Boosting Machine	Training	0.851	0.848	0.763	0.252	0.379	0.109	0.982	0.854
	Testing	0.739	0.825	0.609	0.141	0.230	0.131	0.979	0.835
Neural Network	Training	0.808	0.841	0.642	0.304	0.413	0.115	0.962	0.860
	Testing	0.721	0.816	0.500	0.202	0.288	0.137	0.954	0.841
K-Nearest Neighbors	Training	0.888	0.851	0.765	0.270	0.399	0.104	0.981	0.857
	Testing	0.719	0.821	0.556	0.152	0.238	0.135	0.973	0.835
Support Vector Machine	Training	0.960	0.891	0.980	0.417	0.585	0.085	0.998	0.884
	Testing	0.675	0.810	0.286	0.020	0.038	0.143	0.989	0.817
Gradient Boosting Machine	Training	0.795	0.838	0.685	0.217	0.330	0.120	0.978	0.847
	Testing	0.740	0.823	0.567	0.172	0.264	0.131	0.970	0.838

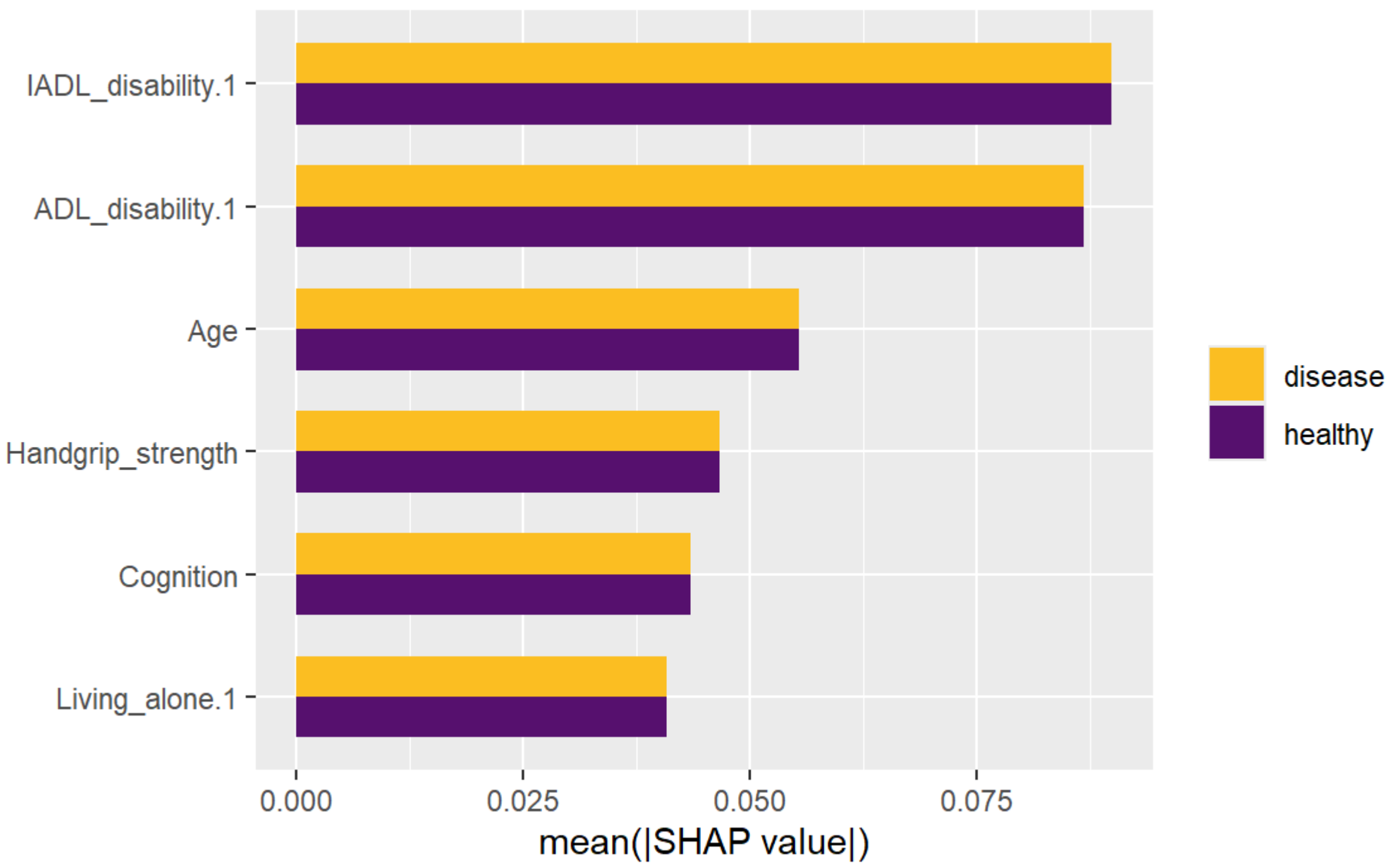

Figure 4 SHAP feature importance bar plot for the Naïve Bayes model

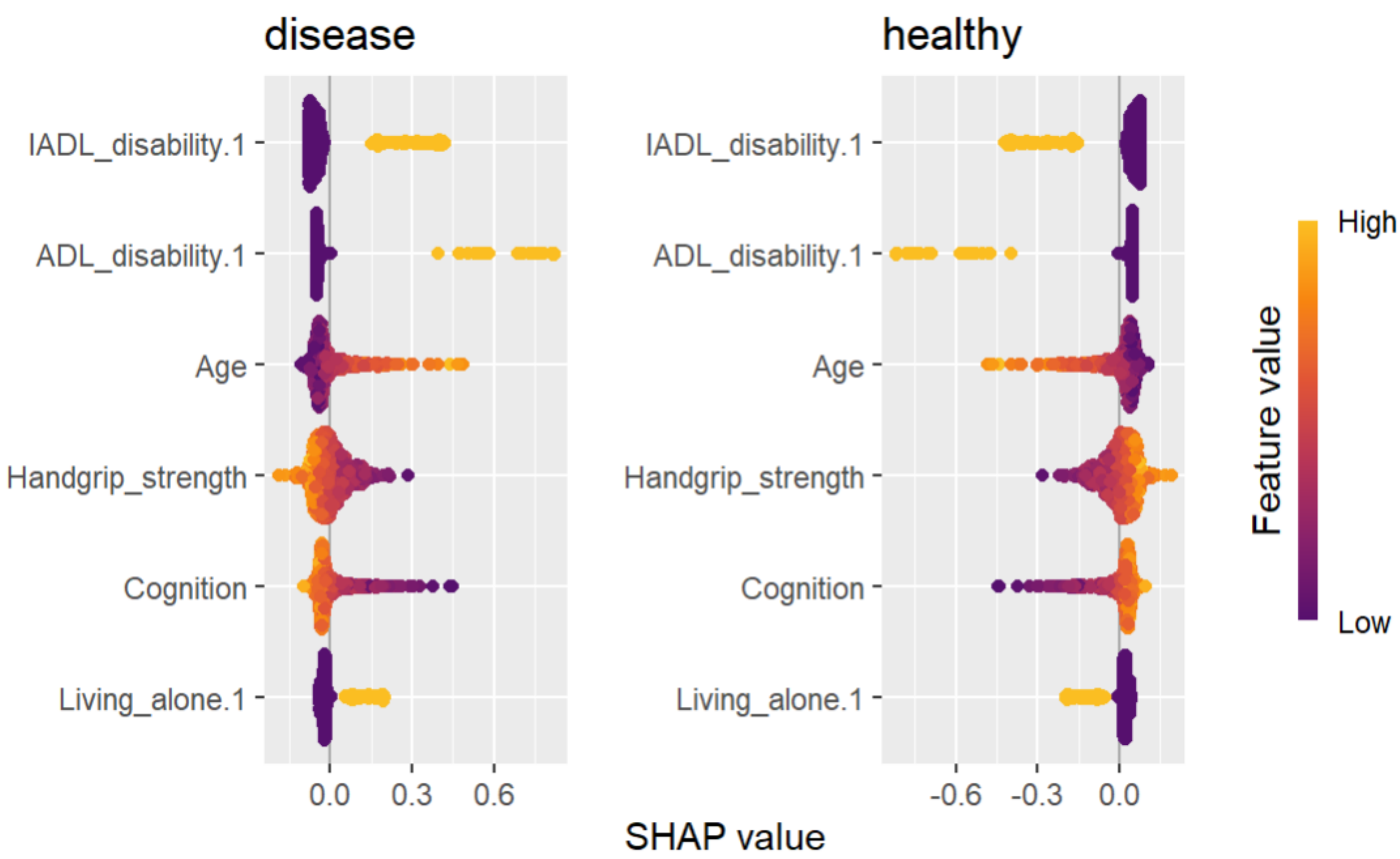

Figure 5 SHAP summary plot for the Naïve Bayes model

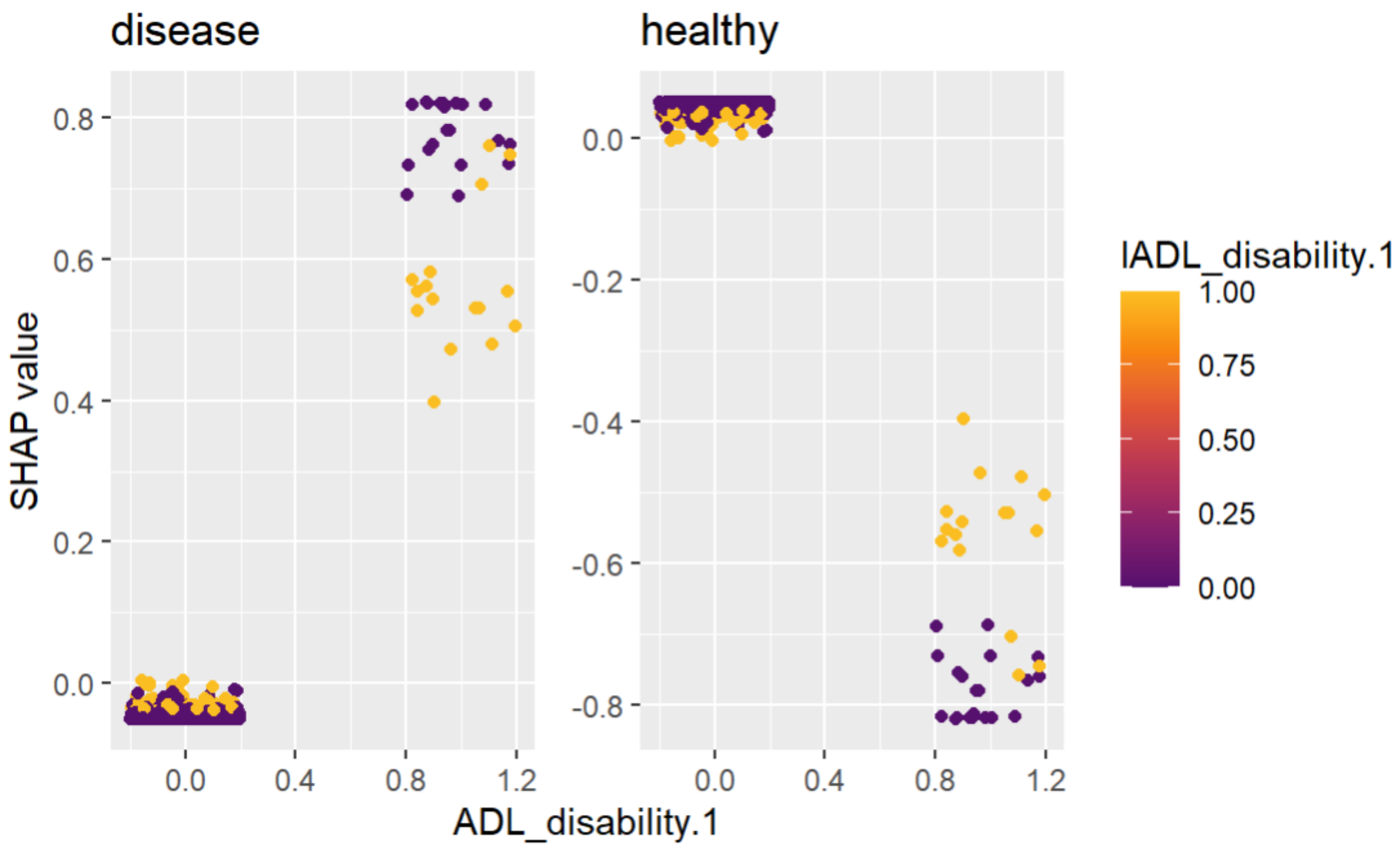

Figure 6 SHAP dependence plot for ADL disability from the Naïve Bayes model

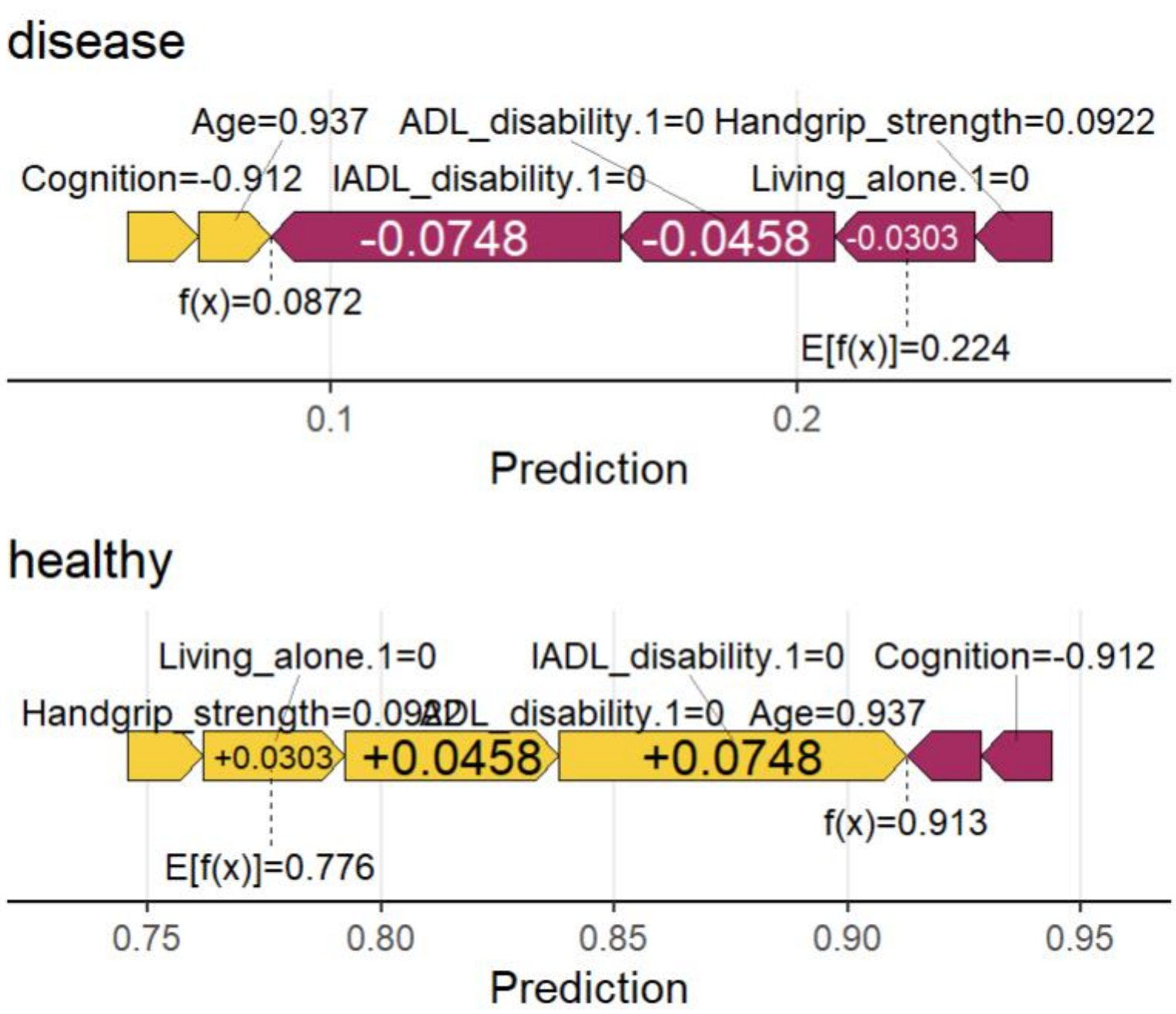

Figure 7 SHAP force plot for the highest-probability sample from the Naïve Bayes model

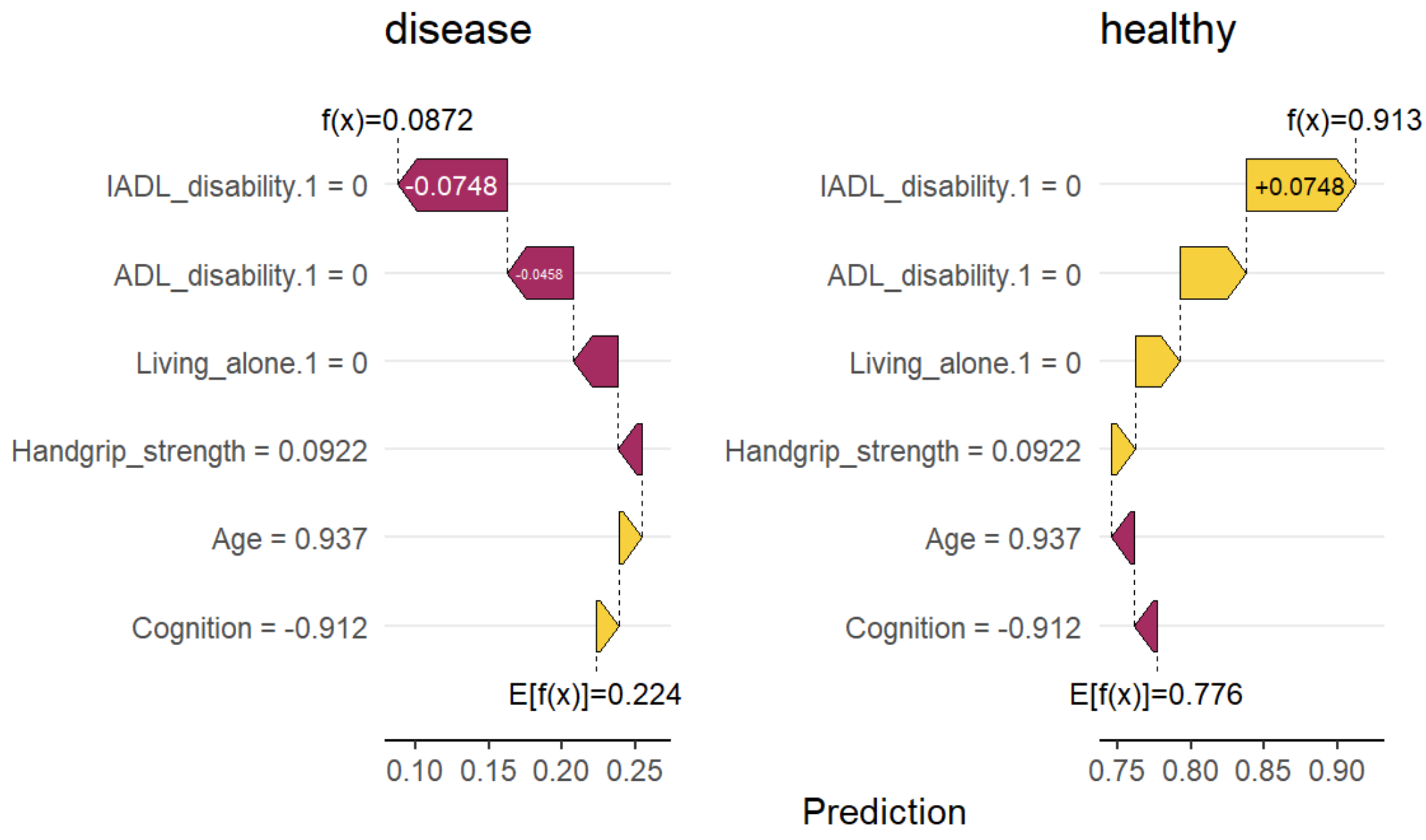

Figure 8 SHAP waterfall plot for the highest-probability sample from the Naïve Bayes model
